# Influence of Ispaghula and Zein Coating on Ibuprofen-Loaded Alginate Beads Prepared by Vibration Technology: Physicochemical Characterization and Release Studies

**DOI:** 10.3390/scipharm86020024

**Published:** 2018-06-05

**Authors:** Jillian Jin Lyn Heng, Jia Hao Teng, Muniyandy Saravanan, Janarthanan Pushpamalar

**Affiliations:** 1 Jeffrey Cheah School of Medicine and Health Sciences, Monash University Malaysia, Jalan Lagoon Selatan, 47500 Bandar Sunway, Selangor Darul Ehsan, Malaysia; jillianjinlyn@gmail.com (J.J.L.H.); jason_90_2@hotmail.com (J.H.T.); 2 Department of Pharmacy, Fatima College of Health Sciences, P.O. Box 24162 Al Maqam, Al Ain, UAE; 3 School of Science, Monash University Malaysia, Jalan Lagoon Selatan, 47500 Bandar Sunway, Selangor Darul Ehsan, Malaysia; pushpa.janarthanan@monash.edu

**Keywords:** alginate beads, microencapsulation, coating, ispaghula, zein, vibration technology

## Abstract

The purpose behind the work was to fabricate alginate beads with better drug loading and extended drug release. Ispaghula was used to enhance the drug loading while zein was employed to extend the drug release. Ibuprofen was employed as a model drug in this study. Ibuprofen-loaded alginate beads with and without ispaghula were prepared using vibration technology and coated with zein. The beads prepared with alginate alone were shown to have loading and entrapment efficiencies of 35% and 70% *w/w*, respectively. Addition of ispaghula in alginate showed a significant increase (*p* < 0.05) in the drug loading (42% *w/w*) and entrapment efficiency (84% *w/w*). Fourier-transform infrared spectroscopy confirmed the presence of ispaghula and zein coating in the alginate beads as well as the ibuprofen loading. Scanning electron microscopy revealed better spherical geometry in the beads with ispaghula. The surface morphology of the uncoated beads was rough due to crystalline and surface drug. The zein coating has produced a smoother surface and particle adhesion. Differential scanning calorimetry has shown a reduction in drug crystallinity. Alginate beads extended the drug release for 4 h and the presence of zein extended the release for 6 h.

## 1. Introduction

Sodium alginate has been extensively used in drug delivery because of its biocompatibility and gel-forming ability with divalent ions [1]. Beads containing drug can be readily prepared by dropping the mixture of drug and alginate into calcium chloride solution (CaCl_2_) [2]. Many investigations were done in alginate beads to get optimum drug loading and release profiles. The required releases were attained using alginate alone or with the mixture of other materials such as carboxymethyl cellulose [3], ispaghula [4], locust bean gum [5] and starch [6] in order to get desired physiochemical properties. One of the key disadvantages is the loss of drug during the crosslinking process with CaCl_2_ solution. As crosslinking progresses, the drug tends to come out from the alginate beads due to shrinkage of gel network [7].

In the present investigation, we have studied the impact of the addition of ispaghula on the physicochemical properties of alginate beads. Ispaghula is a husk obtained from *Plantago ovata*, and is a hydrophilic material capable of retaining water. The ispaghula husk contains mucilage, and its hydrolysis yields d-xylose, l-arabinose, d-galactose and d-galacturonic acid [8]. Though alginate–ispaghula blend matrixes are reported throughout the literature [9,10], there is no clear explanation of the influence of ispaghula on drug loading. We expect that the addition of ispaghula, because of its high viscosity, hydrophilicity and swelling property, will tend to slow down drug loss during crosslinking and enhance drug loading capacity of alginate beads. In addition, the impact of zein coating on the release profile was also studied. Zein is a hydrophobic whey protein, and could be used to coat alginate beads [11,12]. The hydrophobic coating could retard the water uptake and thus be expected to sustain the drug release from the alginate beads. To the best of our knowledge, this is the first time we are reporting the influence of zein coating in the drug release profile of alginate beads. In the present work, drug-loaded alginate beads were prepared using ispaghula and zein coating. Ibuprofen, a nonsteroidal anti-inflammatory drug [2] is used as a model drug in the encapsulation process. The pKa of ibuprofen is reported between 4.9 and 5.2 [13]. It is less ionised in the pH less than 2 and more ionised at pH 6.9 and above.

## 2. Materials and Methods

### 2.1. Materials

Sodium alginate from brown algae and zein from maize were purchased from Sigma-Aldrich, Malaysia. Ispaghula (*Plantago ovata*) was purchased from Sidhpur Sat-Isapgol factory, Sidhpur, Gujarat, India. Ibuprofen was purchased from Lianyungang Zhongyi International Trade Co. Ltd. (Lianyungang, Jiangsu, China). All other chemicals are analytical grade and obtained from RM Chemicals, Malaysia.

### 2.2. Preparation of Ibuprofen-Loaded Alginate Beads

5 g of sodium alginate was dissolved in 250 mL of water using magnetic stirring at room temperature (100 rpm). 5 g of ibuprofen was taken in a mortar; a small amount of alginate solution was added and triturated well to form a lump-free thick paste. More amounts of alginate solution were added (approximately 25 to 30 mL) to make a pourable mixture. While stirring (200 rpm), ibuprofen–alginate mixture was added to the rest of the alginate solution to form a lump-free suspension. The suspension was immediately dripped into 5% *w/v* CaCl_2_ solution using a Buchi encapsulator (B-390, BÜCHI Labortechnik AG, Flawil, Switzerland) fitted with a nozzle (750 µm) and a vibration frequency of 600 Hz with an electrode voltage of 1000 mV. The air pressure was maintained between 500 to 700 mbar to generate beads. A curing time of 30 min was allowed for the crosslinking of the beads. After that, the beads were separated using a nylon mesh and washed with water several times to remove unreacted CaCl_2_ and finally dried at 40 °C until constant weight. Dried beads were kept in glass vials and stored in the desiccator at room temperature.

### 2.3. Preparation of Ibuprofen-Loaded Alginate–Ispaghula Beads

5 g of sodium alginate was dissolved in 150 mL of water using magnetic stirring. 500 mg of ispaghula husk was stirred in 100 mL of water overnight using a magnetic stirrer at room temperature. The viscous solution produced was filtered through a muslin cloth to remove debris and undissolved material. The clear viscous ispaghula solution was added to 150 mL of alginate solution and mixed well. The beads were prepared as explained in the previous section.

### 2.4. Coating with Zein

A 10% *w/v* solution of zein was prepared in 80% *v/v* of ethanol. 1 g of respective beads was placed on a nylon mesh strainer (14 cm diameter) with a holder to produce a uniform layer. 5 mL of zein solution was sprayed in small quantities at the surface of beads using a manual atomizer. The spraying was done to wet the entire surface of the beads and the coating was dried by blowing hot air (40–50 °C) using a conventional hair dryer (Philips, Malaysia). The beads were rotated by gently shaking the strainer and spraying/drying was continued to the completion of 5 mL coating solution in order to produce a uniform coating over the surface of the beads.

### 2.5. Drug Loading and Entrapment Efficiency

Ibuprofen content in the microcapsules was determined via an extraction method. 300 mg of beads was immersed in 100 mL of phosphate buffer (pH 6.8) at room temperature overnight. The solution was subjected to sonication with a Hielscher UIP500hd ultrasonic homogeniser at 600 Hertz amplitude for 1 min to break the beads and dissolve the drug. The resulting solution was filtered using Whatman filter paper No. 40. The filtrates were made up to 100 mL with phosphate buffer (pH 6.8) and estimated with UV–vis spectrophotometer (UV-1800, Shimadzu, Kyoto, Japan) at 264 nm. The theoretical percentage of drug loading (TDL) was calculated using the following equations [13].

TDL = (Weight of drug added (g)/Weight of polymers and drug added (g)) × 100(1)

The percentage of drug entrapment efficiency (DEE) was calculated according to the following equation.

DEE = (Actual drug loading/TDL) × 100(2)

### 2.6. Fourier-Transform Infrared Spectroscopy

The beads were powdered using a mortar and pestle. The infrared spectra of the powdered samples were measured between 600 and 3800 cm^−1^ in a Varian 640-IR FTIR (Agilent, Waltham, MA, USA) spectrophotometer using an attenuated total reflection accessory.

### 2.7. Field-Emission Scanning Electron Microscopy

A field emission scanning electron microscope (SU-8010, Hitachi, Tokyo, Japan) was used to observe surface morphology and the shape of the beads. Cross-section of the beads was done by using a sharp stationary blade after soaking the beads in water for about an hour. The samples were fixed in stubs using double-faced copper adhesive tape and were coated with a thin layer of platinum using a Q150R S rotary-pumped sputter coating system (Quorum Technologies, Lewes, UK) before being observed.

### 2.8. Differential Scanning Calorimetry

Differential scanning calorimetry (DSC) analyses were performed in a temperature range from 50 to 200 °C on a DSC 1 (Mettler Toledo, Greifensee, Switzerland). Approximately 5 mg of each respective sample was weighed and hermetically sealed into the aluminium crucible. The heating rate was 10 °C/min, and the nitrogen flow rate was 50 mL/min.

### 2.9. In-Vitro Release

The release studies were carried out to find the ability of the zein coating to extend the drug release from the beads. Ibuprofen is poorly soluble in acidic pH and hence pH 6.8 is used as the dissolution medium. The dissolution studies were carried out in a USP (United States Pharmacopoeia) dissolution tester (TDT-08L, Mumbai, India) by a paddle method and using 900 mL of phosphate buffer pH 6.8 as the release medium. Beads equivalent to 200 mg of drug were individually filled in hard gelatin capsules. Capsules were dropped in each vessel, and the dissolution rates were measured at 37 ± 0.5 °C and 50 rpm. The samples were withdrawn at regular intervals, and the drug content was estimated at 264 nm using UV-visible spectrophotometer.

### 2.10. Statistical Analysis

Drug loading, entrapment efficiency and drug release data were analysed using repeated-measures one-way ANOVA assuming Gaussian distribution and using the Geisser–Greenhouse correction (GraphPad Prism 7.02 software, La Jolla, CA 92037, USA). The *p*-value of 0.05 was identified as statistically significant. Uncorrected Fisher’s LSD (Least Significant Difference) multiple pairwise comparisons of the data were conducted to determine the statistical significance between the beads.

## 3. Results and Discussion

### 3.1. Preparation and Entrapment Efficiency of Alginate Beads

The preparation conditions were optimised after making several trial batches. The beads prepared with alginate alone were shown to have an entrapment and loading efficiency of 70.3 ± 3.3% and 35.2 ± 1.6% *w/w*, respectively. As shown in Table 1, alginate beads prepared with ispaghula husk has shown significant increase (*p* < 0.05) in the drug loading (41.8 ± 1.2% *w/w*) and entrapment efficiency (83.7 ± 2.4% *w/w*). Further, multiple pairwise comparisons confirmed the significant increase (*p* < 0.05) in drug loading and entrapment efficiency of alginate beads in presence of ispaghula. It could be due to increased viscosity of the system which prevents drug loss during the crosslinking process. A similar effect was reported [14] for the encapsulation of caffeine in alginate–psyllium beads. However, further increase in ispaghula proportion resulted in a thicker solution and failed to generate droplets and beads.

### 3.2. Fourier-Transform Infrared Spectroscopy

**Figure 1:**
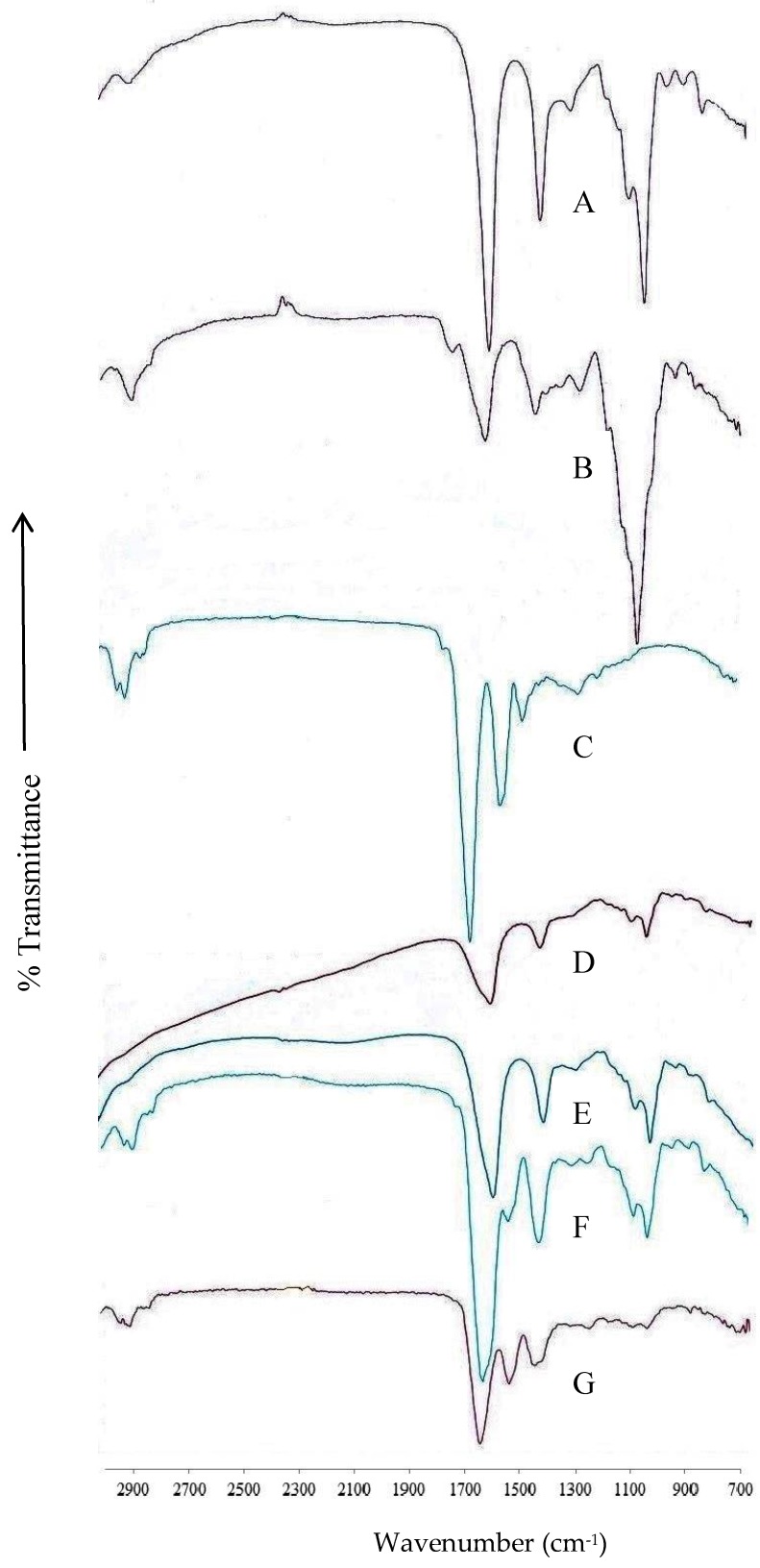
Fourier-Transform Infrared Spectroscopy (FT–IR) spectrum of alginate (**A**); ispaghula (**B**); and zein (**C**); the figure also shows the spectrum of uncoated alginate (**D**) and alginate–ispaghula (**E**) beads without drug. Unloaded alginate (**F**) and alginate–ispaghula (**G**) coated with zein beads also shown for comparison.

The Fourier-Transform Infrared Spectroscopy (FT–IR) spectrum of alginate shows characteristic peaks (Figure 1A) reported throughout the literature [5,14]. Peaks observed at 1606 and 1410 cm^−1^ indicated the stretching vibrations of asymmetric and symmetric C=O in the carboxylic acid groups in alginate molecules. A sharp peak at 1030 cm^−1^ and a small peak at 2922 cm^−1^ reveal C–O stretching and C–H aliphatic stretching vibrations of sodium alginate. Absorption bands of the FT–IR spectrum of ispaghula (Figure 1B) appeared at 2863 cm^−1^ due to CH_3_ symmetric stretching. Strong peaks were observed in the region of 1730 and 1620 cm^−1^ relating to the ester carbonyl and carboxylic groups. Peaks at 1140 cm^−1^ refer to the C–O–H structure in the ispaghula [14]. The bands at 1651 (C=O, amide I) and 1530 (C–N and N–H, amide II) cm^−1^ were observed in the FT–IR of zein, which indicates stretching vibrations of amide groups. Small peaks at 2902 and 2954 cm^−1^ correspond to C–H vibrations within the zein molecule [11,12]. The FT–IR spectra of alginate (Figure 1D) and alginate–ispaghula (Figure 1E) uncoated beads resemble each other, which could be due to the very low proportion of ispaghula, as well as indicating absence of interaction. The intensities of carboxylic acid peaks were reduced in the beads, confirming the crosslinking by calcium ions [12]. The FT–IR spectra of zein-coated unloaded alginate (Figure 1F) and alginate–ispaghula (Figure 1G) were similar. The peaks at 1520, 2902 and 2954 cm^−1^ confirmed the presence of zein coating on the alginate and alginate–ispaghula beads.

**Figure 2:**
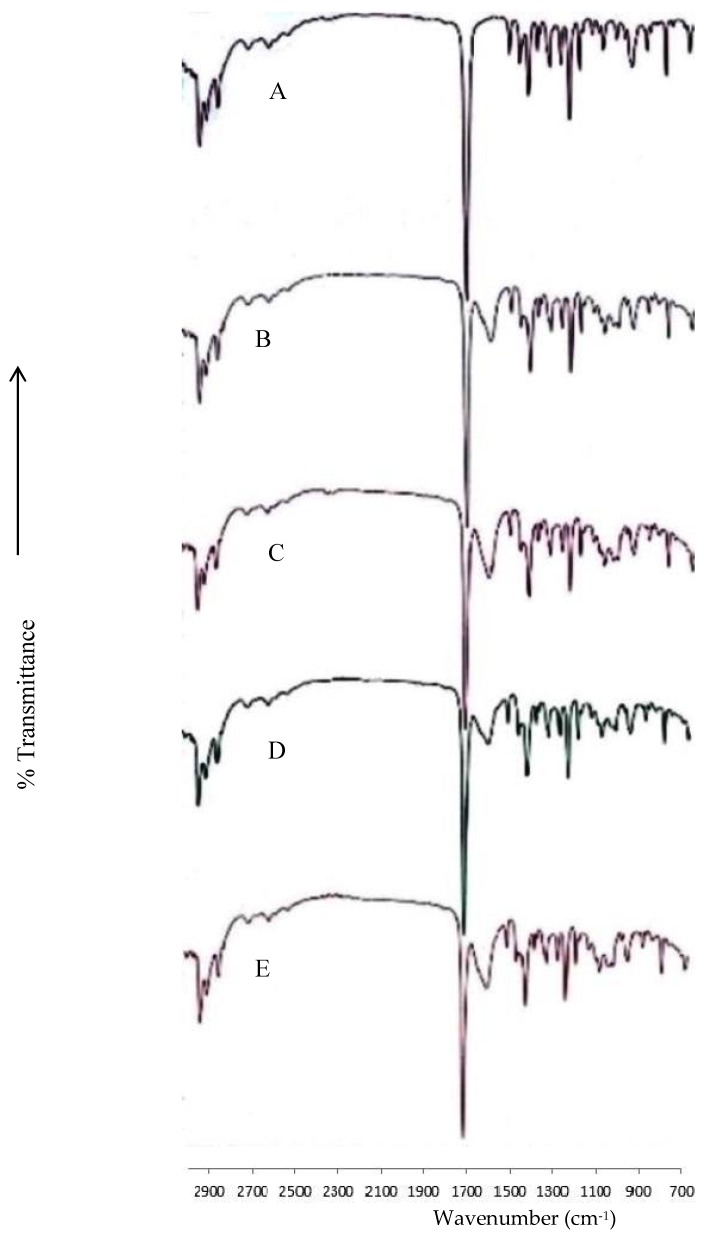
FT–IR spectrum of ibuprofen (**A**); alginate beads loaded with ibuprofen (**B**) and alginate–ispaghula beads loaded with ibuprofen (**C**); the spectrum of zein-coated alginate (**D**) and alginate–ispaghula (**E**) beads loaded with ibuprofen is also shown.

The FT–IR spectrum of ibuprofen (Figure 2A) has shown characteristic peaks of low intensity at 2603 and 2711 cm^−1^, corresponding to the stretching vibration of the cyclic dimerized hydroxyl groups. The bending –OH absorption at 1231 cm^−1^ reveals free hydroxyl groups, and a peak at 1185 cm^−1^ indicates C–O stretching vibrations. Characteristic peaks at 1710 and 2945 cm^−1^ were observed for C=O and –OH stretching, respectively, for the carboxylic acid group in the ibuprofen. The peaks in 1508, 1456 and 780 cm^−1^ are associated with the vibration in the skeleton of benzene. These peaks were in accordance with our previous publication [13]. All these ibuprofen peaks were intact and present in similar intensities in alginate and alginate–ispaghula beads (Figure 2B–E), thus confirming the absence of drug–polymer interaction.

### 3.3. Field-Emission Scanning Electron Microscopy

**Figure 3:**
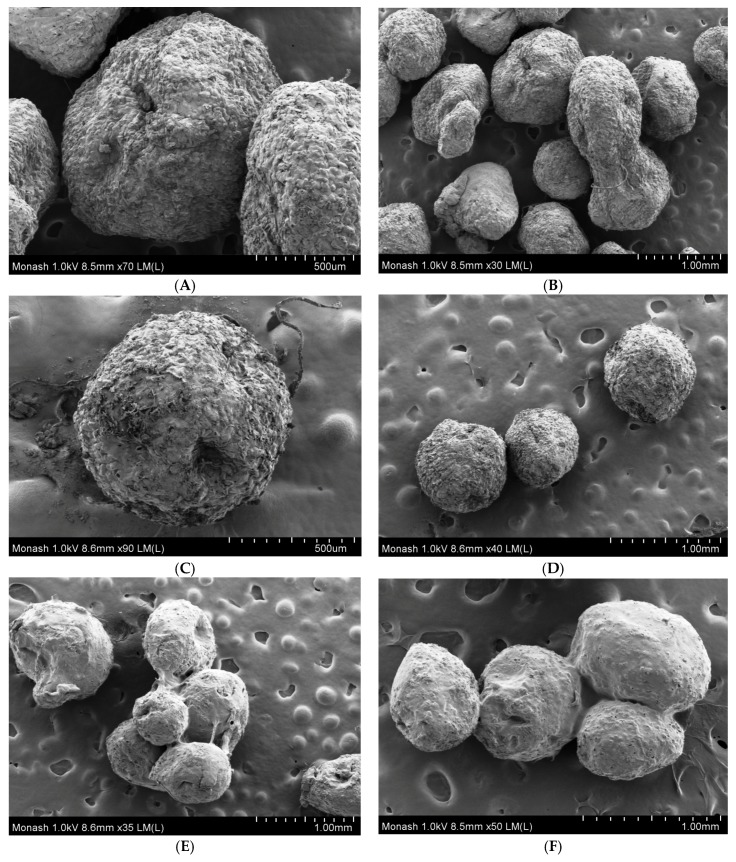
Field-Emission Scanning Electron Microscopy (FE–SEM) pictures of ibuprofen-loaded alginate (**A**,**B**) and alginate–ispaghula beads (**C**,**D**). Coating using zein is evidenced by bead adhesion and smooth surface of ibuprofen-loaded alginate (**E**) and alginate–ispaghula beads (**F**).

**Figure 4:**
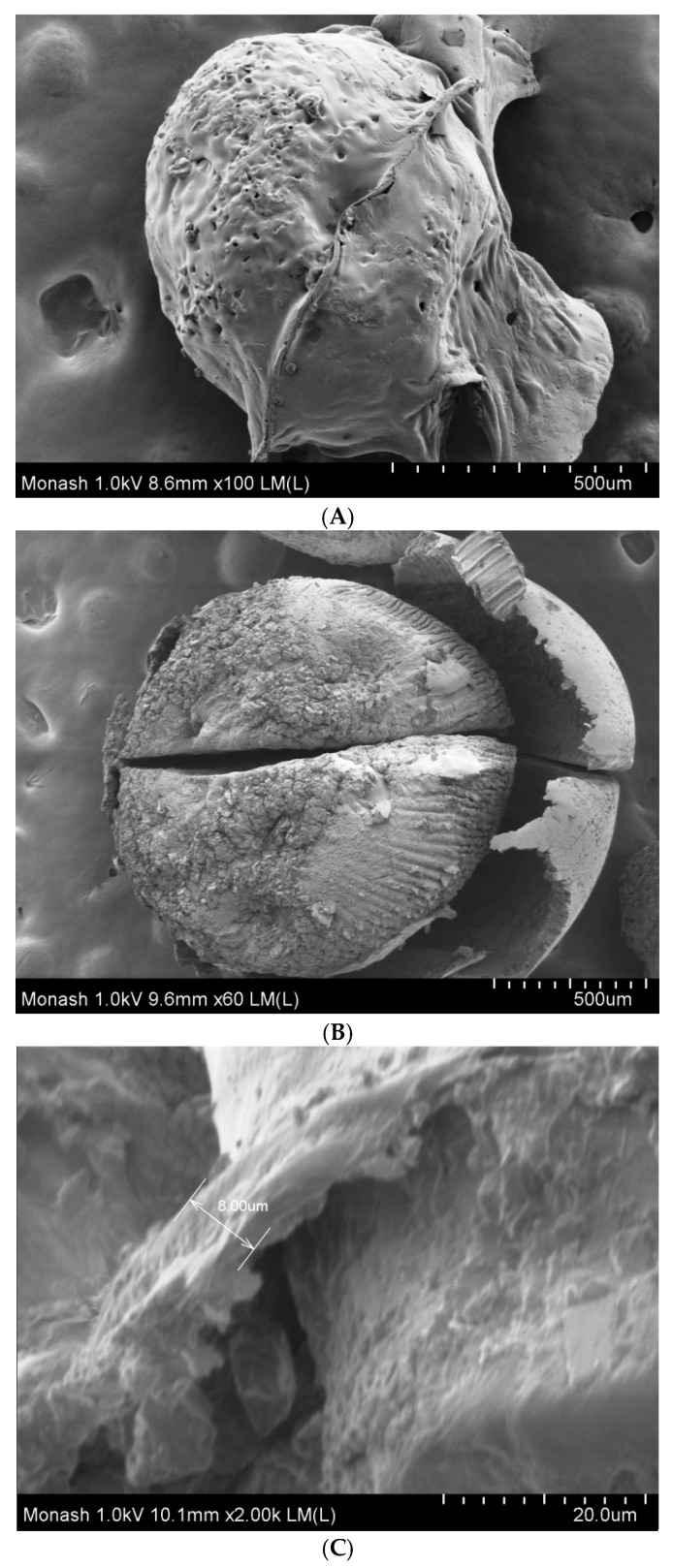
Surfaces of coated ibuprofen beads (**A**) were smooth. Cross-section of the bead clearly reveals formation of zein film (**B**) with a coating thickness of 8 µm (**C**).

The surfaces of ibuprofen-loaded alginate and alginate–ispaghula beads were rough due to the presence of crystalline drugs (Figure 3A–D). The shapes of drug-loaded alginate beads were not spherical which could be due to intensive crosslinking with calcium ions (Figure 3B). In contrast, the alginate–ispaghula beads were spherical and uniform in size (Figure 3D). The coated beads were aggregated (Figure 3E,F) and the surface was smooth (Figure 4A). The coating of zein is evidenced by film formation around the beads (Figure 4A). The cross-section of the bead clearly reveals the presence of zein coating with a thickness of approximately 8–12 µm (Figure 4B,C).

### 3.4. Differential Scanning Calorimetry

**Figure 5:**
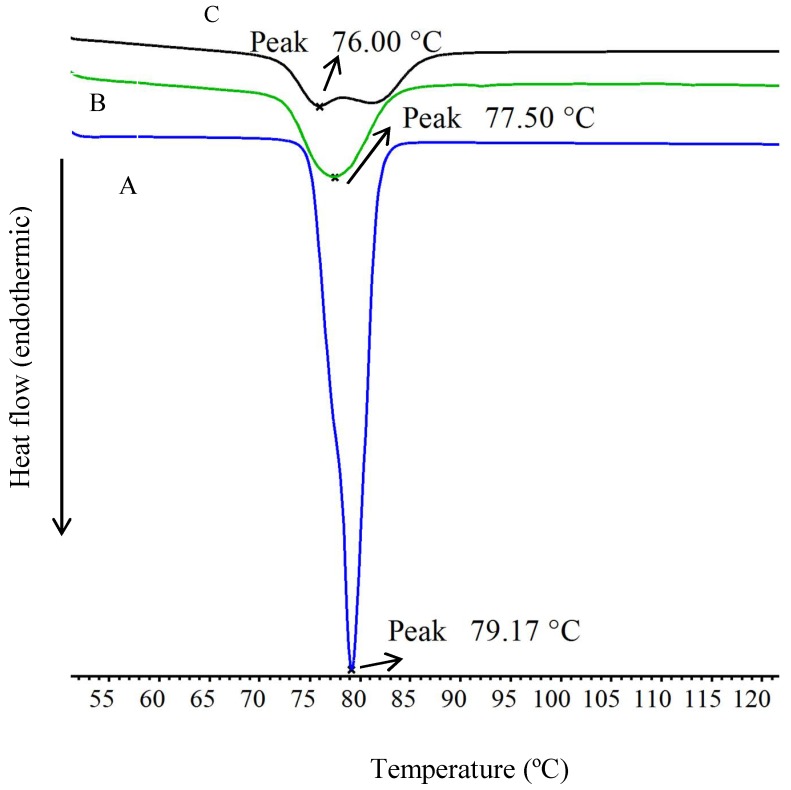
Differential scanning thermograms (DSC) thermograms of ibuprofen (**A**), alginate (**B)** and alginate–ispaghula (**C**) beads loaded with ibuprofen.

DSC thermogram of ibuprofen (Figure 5) has shown a sharp crystalline endothermic peak at 79 °C [13,15] due to melting. The drug-loaded alginate and alginate–ispaghula beads were shown with peaks at 77.5 and 76 °C, respectively, indicating the crystalline and stable nature of ibuprofen. However, the slight reductions in the peak as well as short peaks indicate the reduction in the drug crystallinity (Figure 5). Further, the drug-loaded ibuprofen alginate–ispaghula bead has shown a broader peak than alginate beads, suggesting the presence of less-crystalline or more-amorphous drug.

### 3.5. In-Vitro Release Studies

**Figure 6:**
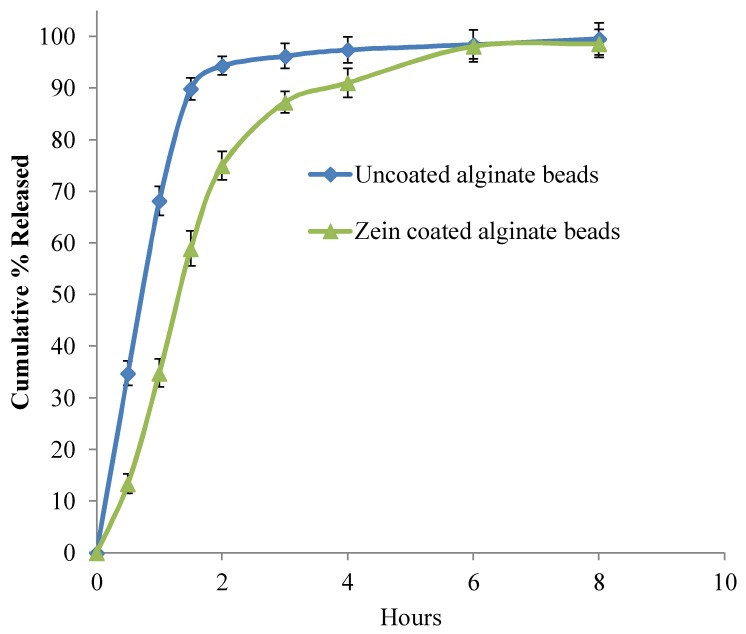
Ibuprofen release from uncoated and zein-coated alginate beads. Each datapoint represents average of three readings, and bar represents standard deviation.

**Figure 7:**
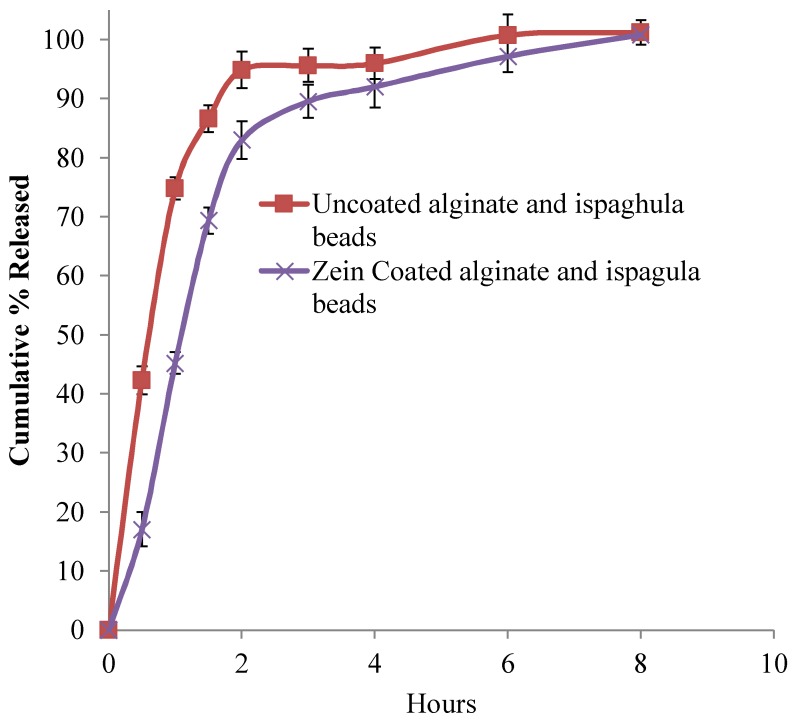
Ibuprofen release from uncoated and zein-coated alginate–ispaghula beads. Each datapoint represents average of three readings, and bar represents standard deviation.

As shown in Figure 6, alginate beads could extend the drug release about 4 h, whereas zein-coated alginate beads sustained the drug release for 6 h. A similar release pattern was observed in uncoated and zein-coated alginate–ispaghula beads (Figure 7). The addition of ispaghula in alginate beads resulted in a relatively faster drug release and could be due to the presence of less crystalline drug as indicated in DSC analysis. Repeated-measures ANOVA showed a significant difference in the release pattern of all formulated beads (*p* < 0.05). Further pairwise comparisons using uncorrected Fisher’s LSD indicated the significant difference between the release pattern of uncoated and coated alginate beads. However, the release profile of alginate and alginate–ispaghula beads has shown insignificant difference (*p* > 0.05).

## 4. Conclusions

Alginate beads are one of the simple ways to encapsulate pharmaceuticals for extended drug delivery. However, alginate beads possess problems such as low drug loading and faster drug release due to their hydrophilic nature. Alteration of surface by hydrophobic materials might be useful in obtaining a slower drug release. An attempt is made in the present investigation to improve drug loading and sustain drug release in the alginate beads. Addition of ispaghula in alginate beads significantly increases the drug loading and entrapment efficiency. This method could be useful in enhancing the drug loading of water-soluble drugs in alginate beads. Further, coating alginate beads using zein could sustain the drug for an extended period.

## Figures and Tables

**Table 1:** Physicochemical parameters of ibuprofen-loaded alginate beads.

Batch No	Alginate (g)	Ispaghula (g)	Ibuprofen (g)	Drug Loading % (*w/w*)		Entrapment Efficiency * (*w/w*)
				Theoretical	Actual *	
1	5	0	5	50	35.2 ± 1.6	70.3 ± 3.3
2	4.5	0.5	5	50	41.8 ± 1.2	83.7 ± 2.4

* Mean ± standard deviation (*n* = 5).
